# Full confluency, serum starvation, and roscovitine for inducing arrest in the G_0_/G_1_ phase of the cell cycle in puma skin-derived fibroblast lines

**DOI:** 10.1590/1984-3143-AR2023-0017

**Published:** 2023-04-21

**Authors:** Luanna Lorenna Vieira Rodrigues, Yasmin Beatriz França Moura, João Vitor da Silva Viana, Lhara Ricarliany Medeiros de Oliveira, Érika Almeida Praxedes, José de Brito Vieira, Sarah Leyenne Alves Sales, Herlon Victor Rodrigues Silva, Maria Claudia dos Santos Luciano, Claudia Pessoa, Alexsandra Fernandes Pereira

**Affiliations:** 1 Laboratório de Biotecnologia Animal, Universidade Federal Rural do Semi-Árido, Mossoró, RN, Brasil; 2 Laboratório de Oncologia Experimental, Universidade Federal do Ceará, Fortaleza, CE, Brasil; 3 Laboratório de Reprodução de Carnívoros, Universidade Estadual do Ceará, Fortaleza, CE, Brasil

**Keywords:** felids, cell cycle synchronization, culture to confluence, somatic cell nuclear transfer

## Abstract

The puma population is constantly decreasing, and cloning by somatic cell nuclear transfer can be used to conserve the species. One of the factors determining the success of the development of cloned embryos is the cell cycle stage of the donor cells. We evaluated the effects of full confluency (~100%), serum starvation (0.5% serum), and roscovitine (15 µM) treatments on the cell cycle synchronization in G_0_/G_1_ of puma skin-derived fibroblasts by flow cytometric analysis. Also, we assessed the effects of these synchronization methods on morphology, viability, and apoptosis levels using microscopy tools. The results showed that culturing the cells to confluence for 24 h (84.0%), 48 h (84.6%), and 72 h (84.2%) and serum starvation for 96 h (85.4%) yielded a significantly higher percentage of cells arrested in the G_0_/G_1_ (P 0.05) phase than cells not subjected to any cell cycle synchronization method (73.9%). Nevertheless, while serum starvation reduced the percentage of viable cells, no difference was observed for the full confluence and roscovitine treatments (P 0.05). Moreover, roscovitine for 12 h (78.6%) and 24 h (82.1%) was unable to synchronize cells in G_0_/G_1_ (P 0.05). In summary, full confluency induces puma fibroblast cell cycle synchronization at the G_0_/G_1_ stage without affecting cell viability. These outcomes may be valuable for planning donor cells for somatic cell nuclear transfer in pumas.

## Introduction

Cloning by somatic cell nuclear transfer (SCNT) has been used as an essential tool in the generation of identical individuals (Gómez et al., 2008), a model for understanding the cellular and molecular aspects involved in nuclear reprogramming (Moro et al., 2015), production of stem cells for use in regenerative medicine (Jun et al., 2019), and conservation strategy for endangered species (Moulavi et al., 2017). In this last application, the success of this technique can already be observed in different groups of species, such as gaur (Lanza et al., 2000), mouflon (Loi et al., 2001), African wild cats (Gómez et al., 2004) and grey wolf (Kim et al., 2007). In large felids, establishing SCNT for the birth of offspring is still challenging, especially in species of the genus *Puma* and *Panthera*. On the other hand, in some individuals, SCNT has allowed the development of somatic resource banks (Praxedes et al., 2019) and the production of cells induced to pluripotency (Verma et al., 2012).

In general, it is well known that the success of this technique in any species is initially identified by the studies of all aspects related to cells donor from nuclei or karyoplasts (Borges and Pereira, 2019). These cells, which in most cases have been fibroblasts derived from the skin of animals (Praxedes et al., 2018), need to be established *in vitro* before being used in SCNT. Three steps are involved in the establishment of nucleus donor cells: (i) isolation and characterization of somatic cells, (ii) establishment of cell lines, and (iii) development of cell cycle synchronization methods in the G_0_/G_1_ phase (Pereira et al., 2019).

We have developed the first two steps in puma (*Puma concolor* Linnaeus, 1781). Recently, we have established somatic tissue banks for the species (Lira et al., 2021), as well as we have established cell lines for their use as nucleus donor cells for cloning (Lira et al., 2022). Considering that there are variations between the synchronization methods of the cell cycle in G_0_/G_1_ among species (Młodawska et al., 2022), including even among felids (Veraguas et al., 2017), we propose to evaluate different culture conditions for puma fibroblasts, aiming to use these cells in G_0_/G_1_.

Three methodologies have been proposed for cell synchronization in G_0_/G_1_. Full confluency is a method that allows cell high-density conditions. The contact surface between adjacent cells gradually increases, leading to contact inhibition causing most cells to stop dividing and remain in the G_0_/G_1_ phase despite the availability of nutrients and growth factors (Curto et al., 2007). Additionally, high-density conditions favor the regulation of reactive oxygen species (ROS) as well as activates coactivator-1α (PGC1α), which functions as a key regulator of energy expenditure, involved in ROS reduction and protection of cells against oxidative stress (Yang et al., 2018). Regarding serum starvation, this methodology act on the checkpoints by depriving the cells of adequate environmental or nutritional conditions (Kues et al., 2000), more specifically acting due to the response to the absence or presence of mitogens to continue the cell cycle during the onset of the G_0_/G_1_ phase, so when these cells are in the absence of mitochondrial growth factors that the serum would offer, they accumulate in a state of a 2n DNA content (Coller, 2007). Finally, roscovitine is a potent aminopurine inhibitor of cyclin-dependent kinase 1 (CDK1/cyclin B), cyclin-dependent kinase 2 (CDK2), and cyclin-dependent kinase 5 (CDK5), thereby synchronizing the cells in G_0_/G_1_ (Gómez et al., 2018).

In this sense, non-activated cytoplasts are high in maturation-promoting factor (MPF) activity, a complex of cyclin B and CDK 1 or p34^cdc2^. When an interphase donor nucleus is introduced into a high MPF milieu, it undergoes nuclear envelope breakdown and premature chromosome condensation (Hashem et al., 2006). Therefore, MPF levels decline following activation, chromatin decondenses, and a nuclear envelope is formed. All nuclei that have undergone nuclear envelope breakdown will then undergo DNA synthesis. Hence, donor nuclei must be in G_0_ or G_1_ when transferred to metaphase II recipient oocytes with high levels of MPF to condense chromosomes normally and maintain the correct ploidy of reconstructed embryos at the end of the first cell cycle (Han et al., 2003). The coordination of the cell cycle between the nucleus of the donor cell and the cytoplasm of the recipient has been widely recognized as a critical factor for the adequate maintenance of integrity and ploidy in SCNT embryos, being necessary for the definition of an adequate method of synchronization of the cell cycle to SCNT success (Nguyen et al., 2021).

The puma is one of seven large felid species in the world and the only one native to the non-tropical regions of the New World (Karandikar et al., 2022), being the fourth largest wild felid and the most widespread native terrestrial mammal of the Americas (LaBarge et al., 2022). These animals are found in diverse habitats and environments, from mountainous temperate regions to tropical areas and from wilderness to areas with high levels of human use (Benson et al., 2020). In this sense, most large carnivore species worldwide have seen significant population declines and range contractions (Wolf and Ripple, 2017), being the most common species in zoos and rehabilitation centers (Echeverry et al., 2020), mostly due to the habitat destruction, which is the main factor responsible for reduction in biodiversity or the total number of species on the planet (Machado et al., 2017).

Despite being considered of Least Concern by the International Union for Conservation of Nature’s Red List of Threatened Species (Nielsen et al., 2015), the puma is considered to be declining in some areas. As a large carnivore intricately linked to other wildlife and habitat associations, from a social and political perspective, its conservation and management present numerous challenges (Nielsen et al., 2015). Most of these species are now legally protected and the focus of worldwide conservation actions (Ripple et al., 2014). Therefore, we aim to compare different cell cycle synchronization methods in G_0_/G_1_ of puma’s fibroblasts to establish this step of the SCNT for the conservation of these animals.

## Methods

The experiments were conducted following the Animal Ethics Committee (CEUA/UFERSA, No. 23091.010755/2019-32), Chico Mendes Institute for Biodiversity Conservation (ICMBio, No. 71804-1) and National System for the Management of Genetic Heritage and Associated Traditional Knowledge (SisGen, No. A420E6F). Unless otherwise stated, the reagents used in this study were obtained from Sigma-Aldrich (St. Louis, USA). Dulbecco’s modified Eagle medium (DMEM) and fetal bovine serum (FBS) were obtained from Gibco-BRL (Carlsbad, USA).

### Establishment of cell lines

Skin tissue samples (1.0-2.0 cm^2^) were obtained from the ear notch of three healthy adult puma, one female and two males, between 2 to 5 years old, at the Ecologic Park Ecopoint (Fortaleza, CE, Brazil) and Sargento Prata Municipal Zoo (Fortaleza, CE, Brazil). The skin samples were cultured, and three fibroblast lines were previously established (Lira et al., 2022). Subsequently, cells from three lines frozen in 10% dimethyl sulfoxide (DMSO), 10% FBS, and 0.2 M sucrose were thawed, and 4^th^ and 5^th^ passage cells were used for this study.

Then, cells were cultured in DMEM supplemented with 10% FBS and 2% antibiotic-antimycotic solution in a humid atmosphere containing 6.5% CO_2_ at 38.5 °C. Before initiating cell cycle synchronization protocols, cells were evaluated for their proliferative activity by obtaining the growth curve and determining the population doubling time (PDT). Cells were seeded in 12-well plates at a 1.0 × 10^4^ cells/mL concentration. Cells from each well were counted at 24 h intervals for up to 216 h of culture. After each interval, the mean cell count was recorded; finally, the cell growth curve was generated, and the PDT was estimated based on these measurements.

### Cell treatments and experimental design

In a series of three experiments, we examined the effect of various culture conditions such as full confluency (FC), serum starvation (SS), and the effect of roscovitine (RSV) on the cycle synchrony of puma skin fibroblasts in different incubation times. In each treatment group, cells without any treatment and with 70% confluence were used as a control (growing cells, GC). All treatments were performed in duplicate for each animal, producing six repetitions for each treatment and each incubation time.

In the first experiment, cells were harvested at 90-100% confluency (full confluency) to monitor the effects of confluency on synchronization. The effects of contact inhibition were monitored after 24, 48, and 72 h of an extended culture of full confluency cells. During the treatment of FC, the culture medium composed of DMEM and 10% FBS was changed every 2 days (Gómez et al., 2018).

In the second experiment, for SS, cells at 70% confluency in DMEM with 10% FBS were cultured in DMEM supplemented with 0.5% FBS for 24, 48, 72, and 96 h. The culture medium was changed every 2 days (Wittayarat et al., 2013).

In the third experiment, treatment with 15 µM RSV was performed for 12 and 24 h of cell culture after the cell confluence reached 70%. After starting treatment (day = 0), the stage of fibroblasts from each animal was analyzed after 12 h and 24 h of exposure to RSV (Wittayarat et al., 2013).

### Cell fixation, staining, and cell cycle analysis

After the treatments, cells were trypsinized, centrifuged at 600×g for 10 min, and resuspended in 1.0 mL of cold 70% ethanol for fixation. The cells were then maintained at -20 °C for 5 days. The fixed cells were washed in PBS for ethanol removal, and each sample was centrifuged at 400×g for 10 min. Subsequently, cells were stained with 5 μg/mL propidium iodide (PI), 50 μg/mL RNase was added, and samples were incubated at 4 °C for 50 min. After that, the samples were analyzed using a Guava Easycyte flow cytometer (Guava Technologies, Stamford, Lincolnshire, United Kingdom).

Data were obtained from 15,000 events from each sample. Histograms of PI fluorescence vs. counts were generated to evaluate the percentages of cells for each cell cycle phase (G_0_/G_1_, S, G_2_/M) as well as the levels of sub-G_0_/G_1_. The proportion of cells in each cell cycle phase and sub-G_0_/G_1_ levels was assessed using MODFIT software version 5.0 (Verity, https://www.vsh.com/products/mflt/index.asp).

### Morphological analysis and viability by cell membrane integrity

Cells were evaluated under an inverted microscope (Nikon TS100, Tokyo, Japan) for cell forms and cytoplasmic extensions (Lira et al., 2022). Moreover, trypan blue exclusion assay determined the percentage of living cells (Lira et al., 2022). Briefly, the cells were stained with 0.4% trypan blue in phosphate-buffered saline (PBS) and counted using a hemocytometer.

### Apoptosis level assessment

Twelve microlitres of dye mixture [2 μg/mL acridine orange and 10 μg/mL ethidium bromide, diluted in 8 μL PBS (pH 7.4)], was mixed gently with 50 μL of cell suspension and put onto a clean microscope slide. The suspension was immediately (dye uptake is very fast) examined in a fluorescence microscope (Olympus BX51TF) under 200× magnification at 480 nm (Leite et al., 1999). A minimum of 300 cells was counted using ImageJ software (National Institutes of Health, Bethesda, Maryland, USA) in every sample, and the percentage of cells recorded in four different groups: viable (V), early apoptotic (EA), late apoptotic (LA), and necrotic cells (N). With V: cells with a uniform light green nucleus; EA: cells in initial apoptosis, with a non-uniform green nucleus; LA: cells in late apoptosis, with a non-uniform bright orange nucleus, and N: necrotic cells, with a uniform orange nucleus (Lira et al., 2022).

### Statistical analysis

Data were expressed as mean ± standard error (one fibroblast line/one repetition) and analyzed using the Graph-Pad software (Graph-Pad Software Incorporation, La Jolla, CA, USA). All results were verified for normality by the Shapiro-Wilk test and homoscedasticity by Levene’s test. Since data did not show a normal distribution, they were arcsine transformed and analyzed by ANOVA followed by the Tukey test. Significance was set at P 0.05.

## Results

**Figure 1:**
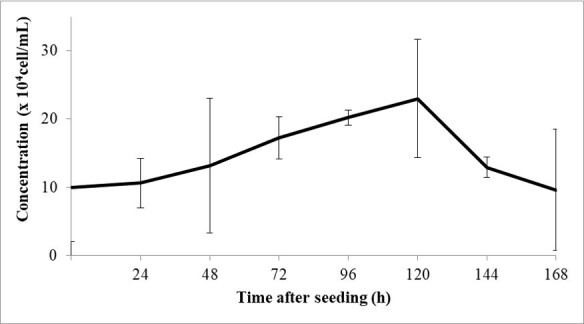
Growth curve of puma fibroblasts before cell treatment for synchronization of cells in G_0_/G_1_. Values are presented in mean ± standard error.

Prior to initiating cell cycle synchronization treatments, thawed cells showed normal morphology with PDT of 53.1 ± 4.1 h. Moreover, cells demonstrated a sigmoidal curve (Figure 1) with the lag phase of adaptation up to day 1 followed by exponential and stationary growth, indicating that these cells were going through various growth phases. Additionally, the decreasing phase was observed.

### Effects of the synchronization methods on cell morphology

**Figure 2:**
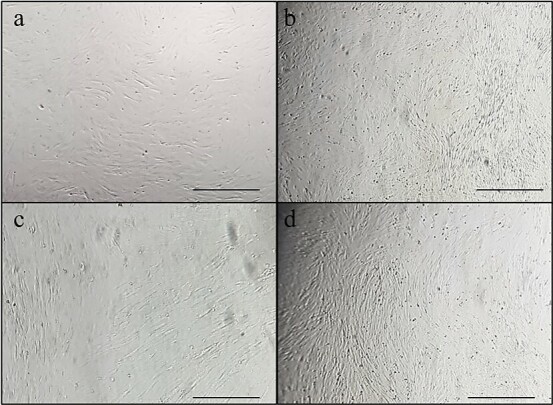
Cell morphology of puma fibroblasts before synchronization (a) and after synchronization by different methods, full confluency (b), serum starvation (c), and exposure to roscovitine (d). Scale bar: 100 µm.

After the three experiments of cell cycle synchronization, cells remained showing normal morphology with a fusiform shape, elongated nucleus, and its extensions, maintaining their characteristics (Figure 2).

### Effects of the synchronization methods on cell viability

**Figure 3:**
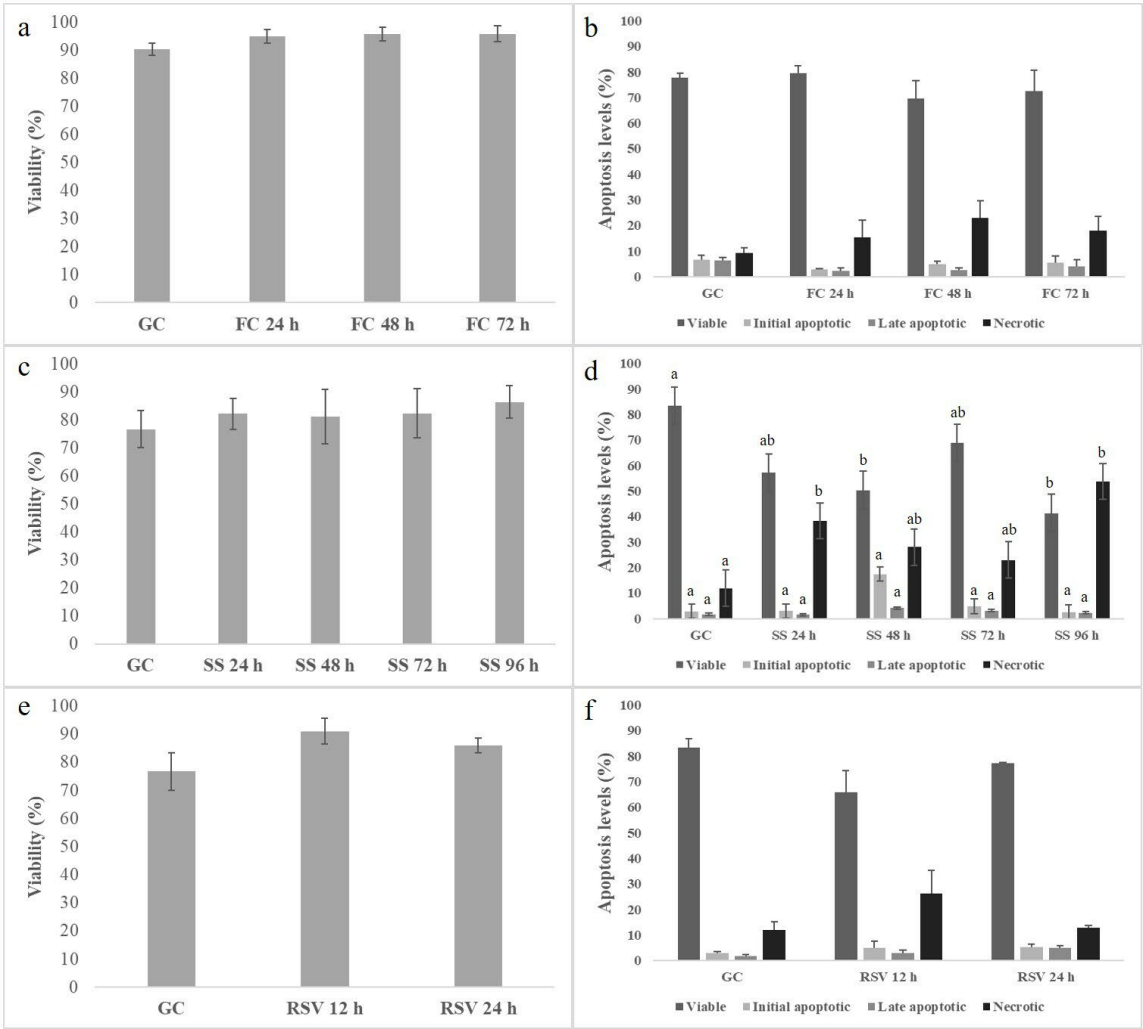
Cell viability (a, c, and e) and apoptosis levels (b, d, and f) in puma fibroblasts subjected to different cell cycle synchronization methods. GC: growing cells; FC: full confluency; SS: serum starvation; and RSV: roscovitine. a,b: indicates differences among parameters (viable, initial apoptosis, late apoptosis, and necrosis) in each treatment (24, 48, 72, 96 h) (P 0.05).

Regarding viability by the trypan blue assay, the FC group was similar to the CG group (90.5% ± 2.2) (P 0.05) at the three times evaluated 24 h (95.1% ± 2.5), 48 h (95.9% 2.4), and 72 h (96.0% ± 2.8) (Figure 3a). The SS group was also similar to its control group (76.8% ± 6.7) (P 0.05) in the four evaluated times: 24 h (82.3% ± 5.5), 48 h (81.2% ± 9.7), 72 h (82.4% ± 8.8), and 96 h (86.5% ± 5.8) (Figure 3c). Finally, regarding the RSV group, there was also no difference (P 0.05) between the two evaluated times (12 h - 91.1% ± 4.5) and (24 h - 86.1% ± 2.6) compared to the CG group (76.8% ± 6.7). (Figure 3e).

### Effects of the synchronization methods on apoptosis levels

In the first experiment, FC for 24, 48 and 72 h did not affect apoptosis levels (V, EA, LA, N) at any of the analyzed intervals (P 0.05, Figure 3b). Regarding to SS, when the apoptosis levels were evaluated by differential staining, SS caused cell damage (P 0.05, Figure 3d). In the third experiment, RSV did not affect apoptosis levels at any of the analyzed intervals (P 0.05, Figure 3f).

### Effects of the synchronization methods on cell cycle stages

**Figure 4:**
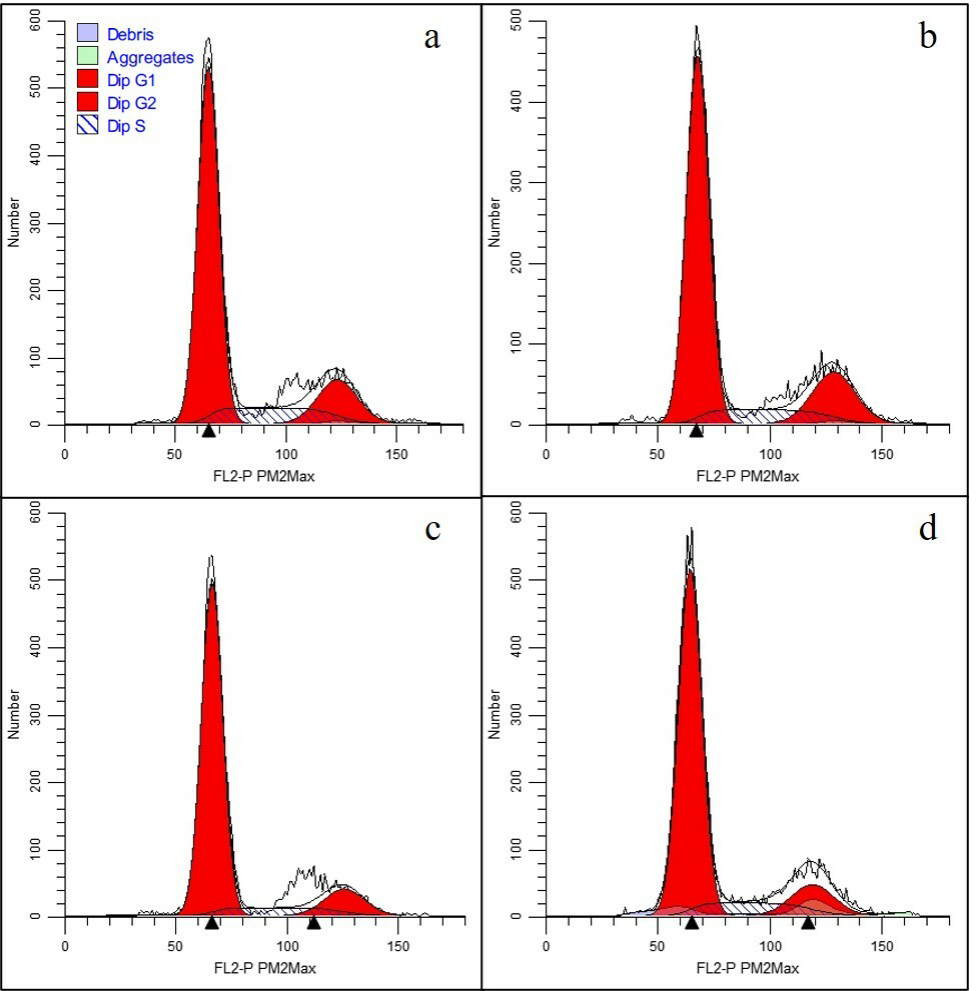
Representative histograms of the flow cytometry analysis of puma fibroblasts. (a) growing cells; (b) full confluency treatment; (c) serum starvation treatment; and (d) roscovitine treatment.

The fibroblasts under the FC treatment for 24, 48, and 72 h significantly increased the proportion of fibroblasts in G_0_/G_1_ phase compared to CG, and the 72-h group decreased the proportion of cells in the S phase (Table 1, Figure 4a-4b). Regarding the G_2_/M phase, FC 24 h decreased the proportion of cells when compared to CG (P 0.05) (Table 1). Also, the three-time intervals evaluated promoted modifications in levels of sub-G_0_/G_1_ (P 0.05). The group of cells on SS for 96 h displayed an increase in the percentage of cells in G_0_/G_1_ (P 0.05) when compared to CG (Table 2, Figure 4a-4c). Additionally, the time of 96 h significantly increased the percentage of G_2_/M and sub-G_0_/G_1_ cells (P 0.05). In the third experiment, it was observed that after 12 and 24 h of RSV treatment, the percentage of G_0_/G_1_, S, G_2_/M, and sub-G_0_/G_1_ was not significantly higher (P 0.05) compared to CG (Table 3, Figure 4a-4d).

**Table 1:** Effect of full confluency (FC) on the percentage of puma fibroblasts in the G_0_/G_1_, S, and G_2_/M phases of the cycle.

**Conditions**	**Cell cycle phase (%)**			
	**G_0_/G_1_**	**S**	**G_2_/M**	**Sub G_0_/G_1_**
**GC**	73.9 ± 3.0^a^	10.4 ± 2.3^a^	15.7 ± 1.2^a^	0.5 ± 0.0^a^
**FC 24 h**	84.0 ± 1.8^b^	7.8 ± 2.3^ab^	8.3 ± 1.1^b^	5.4 ± 1.1^b^
**FC 48 h**	84.6 ± 0.6^b^	3.9 ± 1.2^ab^	11.5 ± 1.2^ab^	12.6 ± 1.6^c^
**FC 72 h**	84.2 ± 1.0^b^	2.3 ± 1.1^b^	13.5 ± 1.1^a^	17.8 ± 1.7^c^

GC: growing cells; FC: full confluency. Values are presented in mean ± standard error. a,b,c: P 0.05.

**Table 2:** Effect of serum starvation (SS) on the percentage of puma fibroblasts in the G_0_/G_1_, S, and G_2_/M phases of the cycle.

**Conditions**	**Cycle cell phase (%)**			
	**G_0_/G_1_**	**S**	**G_2_/M**	**Sub G_0_/G_1_**
**GC**	73.9 ± 3.0^a^	10.4 ± 2.3^a^	15.7 ± 1.2^a^	0.5 ± 0.0^a^
**SS 24 h**	81.3 ± 1.0^a^	6.0 ± 1.0^a^	12.7 ± 0.6^ab^	0.7 ± 0.1^a^
**SS 48 h**	80.4 ± 3.0^a^	6.1 ± 1.4^a^	13.5 ± 1.7^ab^	0.9 ± 0.4^a^
**SS 72 h**	78.4 ± 4.3^a^	6.5 ± 2.6^a^	14.3 ± 1.7^ab^	1.0 ± 0.2^a^
**SS 96 h**	85.4 ± 3.4^b^	6.3 ± 1.5^a^	8.3 ± 2.4^b^	2.3 ± 0.3^b^

GC: growing cells; SS: serum starvation. Values are presented in mean ± standard error. a,b: P 0.05.

**Table 3:** Effect of roscovitine on the percentage of puma fibroblasts in the G_0_/G_1_, S, and G_2_/M phases of the cycle.

**Conditions**	**Cycle cell phase (%)**			
	**G_0_/G_1_**	**S**	**G_2_/M**	**Sub G_0_/G_1_**
**GC**	73.9 ± 3.0	10.4 ± 2.3	15.7 ± 1.2	0.5 ± 0.0
**RSV 12 h**	78.6 ± 3.5	6.6 ± 1.7	14.8 ± 2.1	1.1 ± 0.3
**RSV 24 h**	82.1 ± 4.5	6.5 ± 2.2	11.4 ± 2.5	1.9 ± 0.8

GC: growing cells; RSV: roscovitine. Values are presented in mean ± standard error. P 0.05.

## Discussion

The establishment of karyoplasts as a stage of SCNT is fundamental for developing this technique in a species, especially regarding the synchronization of the cell cycle since the stage of the cycle in which they are found is crucial to guarantee the maintenance of normal ploidy in the embryo reconstructed (Campbell et al., 1996). In this study, we have developed a suitable protocol for somatic cell synchronization in pumas. To our knowledge, this is the first work to compare and evaluate the efficiency of different cycle synchronization methods in G_0_/G_1_ in pumas.

Thus, it was observed that FC for 24 h was more efficient in synchronizing the cell cycle in puma fibroblasts than SS and RSV under the evaluated conditions, as it promoted a greater arrest of cells in the G_0_/G_1_ phase when compared to the other methods. Although no difference was observed for the three times tested (24, 48, and 72 h), we defined the 24 h time as the best because it presented the lowest percentage of cells in sub-G_0_/G_1_, indicating apoptosis, when compared to the CG group. Regarding the viability tests and apoptosis levels, it was observed that the FC treatment at the three evaluated times did not change these parameters, and no difference was observed between the experimental groups and the CG group.

Our findings corroborate what was observed in different studies with wild felids and domestic cats. Gómez et al. (2003) observed that 5 days of FC was more efficient in increasing the percentage of fibroblasts in G_0_/G_1_ of domestic cats (*Felis silvestris catus*) than of African wild cats (*Felis silvestris libica*) (88% vs. 61%, respectively). Song et al. (2007) defined FC (80-90%) as the best methodology for tiger (*Panthera tiger*) fibroblasts. In studies with the Asian golden cat (*Catopuma temminckii*), leopard (*Panthera pardus*), marbled cat (*Pardofelis marmorata*), and Siamese cat, Wittayarat et al. (2013) observed that FC for 5 days promoted an increase of more than 80% in the percentage of cells in G_0_/G_1_ when compared to non-synchronized cells, without an increase in apoptotic cells in all species, except for the marbled cat. Still, in the domestic cat, 3-5 days of FC was effective in inducing a higher percentage of G_0_/G_1_ fibroblasts (~80-85%) compared to growing cells; however, in kodkod (*Leopardus guigna*), FC was efficient after 1- 3 days, but not after 5 days of treatment (Veraguas et al., 2017).

Recently, Młodawska et al. (2022) observed that in Pallas’s cat (*Otocolobus manul*; *Felis manul*) or jaguarundi (*Puma yagouaroundi*; *Harpailurus yagouaroundi*), FC alone did not cause a major change in the proportion of quiescent cells. However, in the domestic cat, FC prolonged more efficiently than the same period of SS at 40-50% confluence. These authors also point out that cell culture to total confluence in jaguarundi can be a valuable method to obtain a high proportion of skin fibroblasts in the G_0_/G_1_ phase without causing damage to the cells, which corroborates our work using cells from animals of the same genus.

As for SS, only the 96-h group increased the proportion of puma fibroblasts in G_0_/G_1_, showing a significant difference from the CG group. The viability test by exclusion by the trypan blue assay did not show significant results, with the cells remaining viable after the time intervals analyzed. This assay is based on assessing plasma membrane integrity and showed that SS does not adversely affect the cell membrane. However, regarding the intracellular levels of apoptosis, it was observed that SS affects this parameter in terms of the percentage of viable cells and necrotic cells. Additionally, we observed that this group increased the proportion of cells in sub-G_0_/G_1_, a negative effect.

Our results regarding SS synchronization differ from what Gómez et al. (2003) observed in the domestic cat and the African wild cat, which on SS for 5 days, generated a higher proportion of skin fibroblasts in the G_0_/G_1_ phase than treatment with FC and RSV. However, these authors observed that SS induced higher DNA fragmentation rates in both species’ fibroblasts. In other felid species, an increasing incidence of apoptosis was observed after 4-5 days of SS for Siamese cat and marbled cat fibroblasts, but not for leopard or Asian golden cat cells (Wittayarat et al., 2013).

Veraguas et al. (2017) observed that in the domestic cat, SS and FC, for both 3 and 5 days, similarly increased the proportion of fibroblasts trapped in the G_0_/G_1_ phase. Nevertheless, SS for 5 days significantly reduced the fibroblast viability of these animals. Still, SS for 3 and 5 days produced the highest proportion of kodkod fibroblasts in the G_0_/G_1_ phase; however, after viability evaluation, only SS for 5 days significantly reduced the fibroblast viability of this wild felid.

Recently, in manul and jaguarundi (Młodawska et al., 2022), the culture of G50 (growing cells at 50% of confluence) and G70 (growing cells at 70% of confluence) under SS conditions resulted in a high proportion of fibroblasts in the G_0_/G_1_ phase, while in the domestic cat, this treatment was efficient only at the G50 confluence. In manul, the fastest effect of SS on the fibroblast cell cycle (after only one day of treatment) was observed at the G70 confluence, while in jaguarundi, at the G50 confluence, demonstrating there is a different response of growing cells to the SS and that this response depends on the level of cell confluence and the treatment duration. These variations between our findings and those of other authors may be due to individual characteristics of the animal (species, breed, sex, and age) from which the cells were obtained, as well as the cell types and culture conditions used (Młodawska et al., 2022).

In the third experiment of this study, we evaluated the efficiency of RSV in fibroblast synchronization and its influence on cell membrane viability and apoptotic levels. The cell membrane remained viable when using RSV at both evaluated times. Furthermore, it was observed that this chemical compound, in the used concentration (15 µM), did not promote cell cycle synchronization in puma fibroblasts, not having differed from the percentage of cells in G_0_/G_1_ of the CG group. For the levels of apoptosis, from our data, it is possible to infer that the concentration of 15 µM does not affect these parameters since these data remained similar to the control group.

Apparently, the effect of RSV depends on the concentration used and the species under study, as shown by the results observed by Wittayarat et al. (2013), where, in the Asian golden cat and the Siamese cat, treatment with RSV greater than 7.5 µM significantly increased the proportion of G_0_⁄G_1_ phase cells compared to the CG group. In contrast, the treatment of marbled cat cells with more than 15 µM of RSV produced the highest percentage of cells in the G_0_⁄G_1_ stage compared to the control groups. However, there was no apparent effect of RSV treatment on the leopard (Wittayarat et al., 2013). Furthermore, it was observed that treatment with 30 µM RSV significantly increased the proportions of apoptotic cells in the Asian golden cat and leopard compared to the control group (Wittayarat et al., 2013).

## Conclusion

In summary, full confluency treatment successfully induces puma fibroblast cell cycle synchronization at the G_0_/G_1_ stage without affecting cell viability. These results may be valuable for planning donor cells for somatic cell nuclear transfer in pumas. Thus, we established the last preparation step for using these fibroblasts as karyoplasts with potential application for conservation.
